# Anti-Inflammatory and Anti-Apoptotic Effects of Acer Palmatum Thumb. Extract, KIOM-2015EW, in a Hyperosmolar-Stress-Induced In Vitro Dry Eye Model

**DOI:** 10.3390/nu10030282

**Published:** 2018-02-28

**Authors:** Yeoun-Hee Kim, Tae Woo Oh, Eunhee Park, Nam-Hui Yim, Kwang Il Park, Won Kyung Cho, Jin Yeul Ma

**Affiliations:** 1 Korean Medicine (KM)-Application Center, Korea Institute of Oriental Medicine (KIOM), 70 Cheomdan-ro, Dong-gu, Daegu 41062, Korea; bigeye38@naver.com (Y.-H.K.); taewoo2080@kiom.re.kr (T.W.O.); black_rica@kiom.re.kr (E.P.); nhyim@kiom.re.kr (N.-H.Y.); kipark@kiom.re.kr (K.I.P.); 2 Institute of Biomedical Engineering Research, Medical School, Kyungpook National University, 90 Chilgokjungang-daero 136-gil, Bukgu, Daegu 41405, Korea

**Keywords:** dry eye, human corneal epithelial cells, hyperosmolar stress, anti-inflammation, anti-apoptosis, natural substance, orientin, isoorientin, vitexin

## Abstract

The aim of this study was to assess the anti-inflammatory and anti-apoptotic effects of KIOM-2015EW, the hot-water extract of maple leaves in hyperosmolar stress (HOS)-induced human corneal epithelial cells (HCECs). HCECs were exposed to hyperosmolar medium and exposed to KIOM-2015EW with or without the hyperosmolar media. Tumor necrosis factor (TNF)-α, interleukin (IL)-1β, and IL-6 production and apoptosis were observed, and the activation of mitogen-activated protein kinases (MAPKs) including extracellular signal regulated kinase (ERK), p38 and c-JUN N-terminal kinase (JNK) signaling and nuclear factor (NF)-κB was confirmed. Compared to isomolar medium, the induction of cell cytotoxicity significantly increased in HCECs exposed to hyperosmolar medium in a time-dependent manner. KIOM-2015EW-treatment significantly reduced the mRNA and protein expression of pro-inflammatory mediators and apoptosis. KIOM-2015EW-treatment inhibited HOS-induced MAPK signaling activation. Additionally, the HOS-induced increase in NF-κB phosphorylation was attenuated by KIOM-2015EW. The results demonstrated that KIOM-2015EW protects the ocular surface by suppressing inflammation in dry eye disease, and suggest that KIOM-2015EW may be used to treat several ocular surface diseases where inflammation plays a key role.

## 1. Introduction

Dry eye syndrome (DES) is a common ocular surface disease attributable to disorders of the tear film and ocular surface [1]. Inadequate tear secretion and increased tear evaporation are the two major causes of DES. The two common mechanisms underlying the pathogenesis of ocular surface injury in DES are ocular surface inflammation and increased tear hyperosmolarity [1,2,3,4,5,6]. The following evidence has related ocular surface inflammation with DES: (1) elevated expression and production of pro-inflammatory cytokines (tumor necrosis factor (TNF)-α, interleukin (IL)-1β, IL-6, and interferon (IFN)-γ) in both dry eye patients and experimental dry eye models [7,8,9,10,11]; and (2) effectiveness of anti-inflammatory therapy in treating DES.

An in vitro dry eye model induced by hyperosmotic stress (HOS) has been developed to investigate DES [4,12,13,14]. HOS can result from decreased tear secretion or increased tearevaporation [1,15]. HOS is considered a key pathogenic factor that initiates ocular surface inflammation and apoptosis in dry eye patients, dry eye mouse models, and in vitro hyperosmotic culture models of human cornea epithelial cells (HCECs) [4,16,17,18,19,20]. HOS in tears can damage the surface epithelium, which can trigger the production of signaling molecules, including interleukin, tumor necrosis factor, and matrix metalloproteinases [14,19,21,22].

Increased epithelial apoptosis has also been implicated in DES pathogenesis [19,23,24,25,26]. Exposure of the ocular surface to a hyperosmotic environment causes an imbalance between the extracellular and intracellular compartments, resulting in a net efflux of water from the ocular surface epithelial cells, leading to cell shrinkage [14,27,28]. HOS extracts water from the cell, inducing cell shrinkage. To restore cell volume, cells undergo a process of regulated volume increase within several minutes by the uptake of inorganic ions and water [29,30,31]. Cell shrinkage and increased ionic strength alters cell architecture, denatures proteins, and disturbs cell function [32,33,34]. Persistent HOS induces DNA damage, cell cycle arrest, and apoptosis [32,35].

Mitogen-activated protein kinases (MAPKs), including extracellular signal regulated kinase (ERK), c-Jun N-terminal kinases (JNK), and p38 MAPK, are conserved signaling molecules [36,37,38]. The activated kinases initiate a cascade of protein phosphorylations, involving multiple kinases and nuclear transcription factors such as nuclear factor (NF)-κB, activator protein (AP)-1 and activating transcription factor (ATF) [39,40], which promote the expression of inflammatory cytokines, chemokines, and matrix metalloproteinases. We hypothesized that HOS causes inflammation by activating MAPK signaling in the human corneal epithelium. Here, we investigated the effects of HOS on the activation of MAPK signaling and the production of pro-inflammatory cytokines and chemokines by HCECs.

Clinical evidence has indicated that anti-inflammatory therapies that inhibit inflammatory mediators reduce the symptoms of DES [41]. Commonly used anti-inflammatory drugs such as topical corticosteroids and cyclosporine A (CsA) are available for the treatment of DES [42,43]. Several randomized trials have demonstrated that short-term topical corticosteroid use (up to four weeks) significantly improves the symptoms of DES [41]. However, the potential of adverse effects limits its long-term use [1]. Recently, topical CsA, an immunomodulatory agent, was demonstrated to be effective against DES [1]. However, drugs that are more effective are required.

KIOM-2015EW is a natural-substance-derived and hot-water extract from the *Aceraceae (Acer) palmatum* Thumb. This plant is used in traditional Chinese medicine for alleviating pain, detoxification, treating lumbodorsal carbuncles and ulcerative carbuncles with twigs and leaves and expelling wind, relaxing muscles, stimulating blood circulation with roots, bark, twigs andleaves [44]. Even though they are commonly found and used plants, the effects of *Acer palmatum* Thumb. on eye disease have not been reported; its anti-inflammatory effects have not been investigated in dry eye disease. Therefore, the present study is designed to investigate the potential value of KIOM-2015EW in treating dry eye disease using an in vitro hyperosmolar-stress-induced dry eye model.

In this study, we found that inflammatory cytokines induced by hyperosmolar stress were controlled by KIOM-2015EW, leading to the prevention of HCEC apoptosis. These findings indicate that KIOM-2015EW may have anti-apoptotic and anti-inflammatory roles affecting hyperosmolar stress on the ocular surface.

## 2. Materials and Methods

### 2.1. Materials and Reagents

Dulbecco’s modified Eagle’s medium: Nutrient Mixture F-12 (DMEM/F-12) was purchased from Gibco BRL (Gaithersburg, MD, USA). Fetal bovine serum (FBS), penicillin, streptomycin, and peroxidase-conjugated secondary antibodies were purchased from Hyclone (Logan, UT, USA). 3-[4,5-Dimethylthiazol-2-ly]-2,5-diphenyl-tetrazolium bromide (MTT), ITS (5 μg/mL insulin, 5 μg/mL human transferrin, 5 ng/mL selenium) and Fluorescence mount solution were purchased from Sigma Chemical Co. (St. Louis, MO, USA). 4′, 6-Diamidino-2-phenylindole (DAPI) was purchased from Invitrogen (Carlsbad, CA, USA). A cell counting kit-8 (CCK-8) was purchased from Dojindo Laboratories (Kumamoto, Japan). SP600125, SB203580, and PD98059 were obtained from Calbiochem (San Diego, CA, USA). Anti-TNF-α and anti-IL-6 were purchased from Abcam (Cambridge, MA, USA). Anti-β-actin and IL-1β were purchased from Santa Cruz Biotechnology Inc. (Santa Cruz, CA, USA). Bcl-2, caspase-3, poly (ADP-ribose) polymerase (PARP), p38, p-p38 (Thr180/Tyr182), extracellular regulated kinase (ERK)1/2, p-ERK1/2 (Thr202/Tyr204), c-Jun N-terminal kinase (JNK), and p-JNK (Thr183/Tyr185) were purchased from Cell Signaling Technology (Danvers, MA, USA). Cyclosporine A (CsA) and fluoremetholone (FML) were obtained from Hanlim (Tsporin eye drops 0.05% CsA; Fumelone eye drops 0.6 mL; Hanlim Pharm. Co., Seoul, Korea). Standard compounds were purchased from Sigma (St. Louis, MO, USA).

### 2.2. Preparation of Herbal Extract, KIOM-2015EW

*Acer palmatum* Thumb. leaves purchased from Korea Medicine Herbs Association (Yeongcheon, Korea), were confirmed by Professor Ki Hwan Bae of the College of Pharmacy, Chungnam National University (Daejeon, Korea), and then stored at the herbal bank of KIOM. To prepare KIOM-2015EW, dried *Acer palmatum* Thumb. leaves (1700 g) were ground into a fine powder, KIOM-2015EW formula was soaked in 17 L distilled water and then heat-extracted in an extractor (Cosmos-600 Extractor, Gyeonseo Co., Inchon, Korea) for 3 h at 115 °C, filtered using standard testing sieves (150 µm, Retsch, Haan, Germany), and then concentrated to dryness in a lyophilizer. KIOM-2015EW powder (50 mg) dissolved in 1 mL distilled water was kept at −20 °C prior to use after filtration through a 0.22 µm disk filter.

### 2.3. HCEC Culture

A HCEC (2.040 pRSV-T) line was purchased from the American Type Culture Collection (Manassas, VA, USA). Cells were maintained in DMEM/F12 containing 10% FBS (Gibco), ITS (5 μg/mL insulin, 5 μg/mL human transferrin, 5 nM selenium; Sigma) and 1% penicillin/streptomycin. Cultures were incubated at 37 °C/5% CO_2_, and the medium was changed daily. Sub-culturing was performed when the cell layers were confluent (2–3 days).

### 2.4. Cell-Viability Assay

Cell viability was evaluated by the CCK-8 and MTT assays. Cells (7 × 10^3^/well) were inoculated in a 96-well plate and treated with KIOM-2015EW for 24 h or 450 mOsM media for the specified time. Cell viability assays were performed as described previously [45]. After incubation, cell viability was determined using MTT colorimetric and CCK-8 assays. Color development was measured at 450 for CCK-8 or 560 nm for MTT using a microplate reader (SpectraMax i3, Molecular devices, CA, USA).

### 2.5. ELISA

HCECs were seeded into a 100-mm^2^ dish (density, 1.5 × 10^6^ cells). Following overnight incubation, cell lines were exposed to each of the conditions described in the following section, and incubated with KIOM-2015EW in serum-depleted DMEM/F-12. After 24 h, culture supernatants were collected, centrifuged to pellet any detached cells, and measured using a commercial enzyme-linked immunosorbent assay (ELISA) kit (BD Pharmingen, San Diego, CA, USA) according to the manufacturer’s instructions.

### 2.6. RNA Isolation and Reverse Transcription–Polymerase Chain Reaction (RT-PCR) Analysis

Total RNA was extracted using an RNA extraction solution (BioAssay Co., Daejeon, Korea) and reverse transcribed to cDNA using a 1st Strand cDNA synthesis kit (BioAssay Co., Daejeon, Korea), according to the manufacturer’s protocol. cDNA aliquots were amplified by PCR using the specific primers in Table 1. PCR products were electrophoresed on 1% agarose gels and visualized by GreenLight (BioAssay Co., Daejeon, Korea). The following PCR conditions are indicated in Table 1.

### 2.7. Cellular Protein Extraction and Western Blotting

Protein extraction and western blotting methods were performed as described previously [45]. Membranes were incubated with the following diluted (1:1000) primary antibodies; PARP, Caspase-3, Cox-2, Bcl-2, p-p38, p38, ERK, pERK, JNK, pJNK, and β-actin in Tris-HCl-based buffer containing 0.2% Tween 20 (TBS-T; pH 7.5). Band intensities were measured using ImageJ (US National Institutes of Health, Bethesda, MD, USA) and were normalized to β-actin or total p38, ERK and JNK.

### 2.8. Immunofluorescence Staining

Harvested cells were fixed with 10% neutral-buffered formalin solution at room temperature for 10 min. Cells were permeabilized with 0.3% Triton X-100 for 15 min, and blocked overnight with 5% normal goat serum and bovine serum albumin (BSA) in TBS at 4 °C. Cells were then incubated with monoclonal or polyclonal antibodies against TNF-α, IL-6, and IL-1β (diluted 1:50–100) and further incubated with the appropriate Alexa Fluor 488- or 555-conjugated secondary antibody at room temperature for 1 h; nuclei were then counterstained with DAPI (1 mg/mL). Slides were mounted with Fluorescence mount, and images were captured using a Nikon fluorescence microscope NIS-Elements microscope imaging software (Nikon, Tokyo, Japan).

### 2.9. Flow Cytometry

An fluorescein isothiocyanate (FITC) Annexin V Apoptosis Detection Kit I (BD Biosciences, San Jose, CA, USA) was used to detect cell death. Briefly, after treatment with vehicle, HOS, or HOS + KIOM-2015EW for 24 h, cells were trypsinized and resuspended in binding buffer (0.1 M HEPES/NaOH pH 7.4, 1.4 M NaCl, and 25 mM CaCl_2_). Annexin V-FITC (5 μL) and propidium iodide (PI; 5 μL) were added and incubated for 15 min at room temperature in the dark. Cells were analyzed by flow cytometry (FACSCalibur, Becton Dickinson, CA, USA).

### 2.10. Cytoplasmic and Nuclear Protein Extraction

Proteins from the cytoplasm and nucleus were separated by using NE-PER Nuclear and Cytoplasmic Extraction Reagents (#78835, Thermo scientific, Waltham, MA, USA). Briefly, HCECs were harvested with trypsin- ethylenediaminetetraacetic acid (EDTA) and washed twice with PBS. Then, cells were centrifuged at 16,000× *g* for 5 min, and the supernatants were removed. Ice-cold CER-I and -II solutions were added per the manufacturer’s instructions to separate the cytoplasmic proteins from the nuclear-compartment proteins. Western blotting for β-actin and phospho-IκB-α was performed to ensure that there was no contamination.

### 2.11. High Performance Liquid Chromatography (HPLC) Analysis

The HPLC system (Dionex Co., Sunnyvale, CA, USA) was composed of ultimate 3000 series a binary pump, an auto-sampler, a column oven and a diode array ultraviolet–visible (UV/VIS) detector (DAD). Data acquisition was performed using the Dionex Chromelon. Chromatographic separation was achieved on a Xbridge^®^ C18 column (5 μm, 4.6 × 250 mm, Waters Co., Milford, MA, USA) using trifluoroacetic acid (TFA) water (0.1%, *v*/*v*); solvent A and acetonitrile; solvent B as mobile phase at a flow rate of 1mL/min. The HPLC elution condition was optimized as follows: 0–3 min, 5% B; 3–5 min, 5–10% B; 5–15 min, 10–15% B; 15–35 min, 15–22% B; 35–50 min, 22–45% B. The column oven and auto-sampler injection volume were wet to 40 °C and 10 μL, respectively. The detected wavelength was set at 280 nm and total run time was 50 min. The KIOM-2015EW was extracted in 100% methanol (10 mg/mL) by ultrasonic for 30 min. The standard compounds, including orientin, isoorientin, and vitexin, were dissolved in 100% methanol (1 mg/mL). All working solutions were filtered through a 0.2 mm syringe membrane filter from Whatman Ltd. (Maidstone, UK) before injection into the HPLC-DAD system.

### 2.12. Statistical Analysis

Analyses were performed using GraphPad PRISM^®^ (GraphPad PRISM software Inc., Version 5.02, San Diego, CA, USA) and SPSS version 23 software (IBM SPSS Statistics for Windows, Version 23.0, Armonk, NY, USA). The nonparametric Mann–Whitney U test was performed to show the statistical significance (*p* < 0.05) between the controls (cells untreated with HOS) and the cells exposed to HOS or between the cells treated with/without KIOM-2015EW. Results are expressed as means ±  standard error of the mean (SEM), and *p*  <  0.05 was considered significant.

## 3. Results

### 3.1. Effects of Hyperosmotic Medium and KIOM-2015EW on HCECs

**Figure 1:**
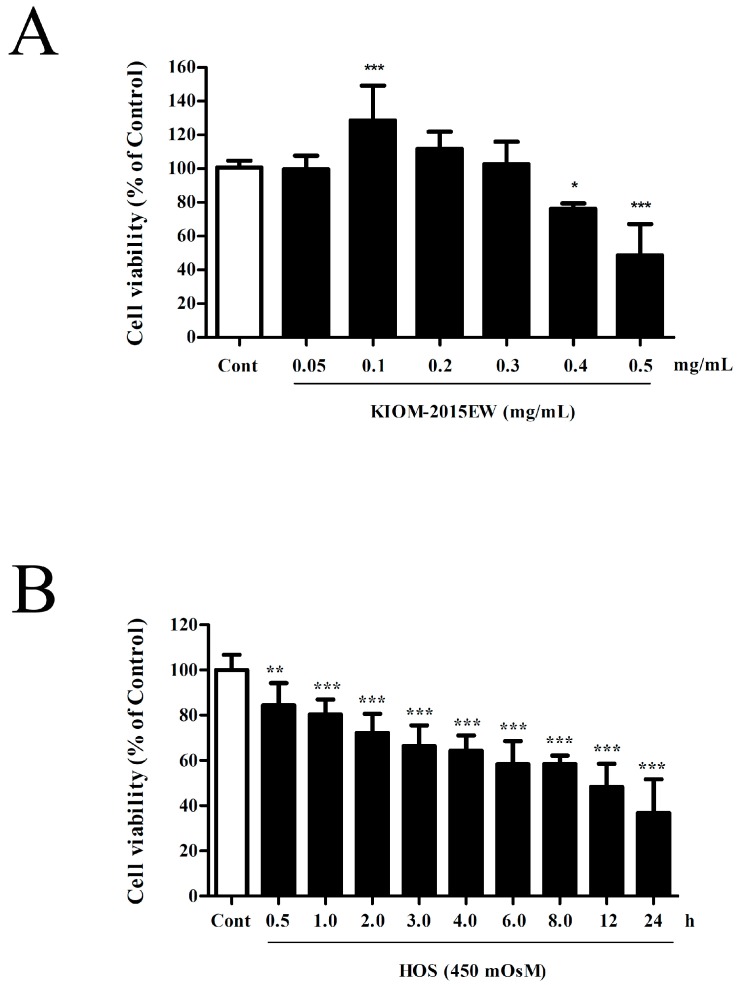
The effects of KIOM-2015EW or hyperosmolar stress (HOS) on cell viability in human corneal epithelial cells (HCECs). (**A**) HCECs were treated with the indicated dose of KIOM-2015EW for 24 h, and cell viability measured by MTT (3-(4,5-Dimethylthiazol-2-yl)-2,5-Diphenyltetrazolium Bromide) assay; (**B**) HCECs were exposed to 450 milliosmoles (mOsM) F-12/ Dulbecco’s Modified Eagle Medium (DMEM) serum-free media for the indicated time, and cell viability measured by cell counting kit (CCK)-8 assay. Data are representative of three independent experiments performed in triplicate. Data are summarized as mean ± standard deviation (SD) from three independent experiments. * *p* < 0.05, ** *p* < 0.01, and *** *p* < 0.001 vs. control.

To determine the effects of KIOM-2015EW-treatment on cell viability, HCECs were seeded in 96-well plates (7 × 10^3^ cells/well), cultured for 24 h, and exposed to KIOM-2015EW at concentrations ranging from 0.05–0.5 mg/mL for 24 h. KIOM-2015EW-treatment had no significant effect on HCEC viability at concentrations up to 0.3 mg/mL. Higher concentrations of KIOM-2015EW (0.4–0.5 mg/mL) decreased cell viability (Figure 1A). Therefore, we used concentrations between 0.05–0.2 mg/mL in subsequent experiments.

To evaluate the effect of HOS-induced cytotoxicity in HCECs, cells were seeded in 96-well plates (7 × 10^3^ cells/well), cultured for 24 h, and then switched to hyperosmolar media for the specified times. Cell viability decreased in a time-dependent manner in cells exposed to 450 mOsM DMEM/F-12 (Figure 1B). Based on the results of these cytotoxicity studies, we subjected cells to 4 h and 24 h of HOS in subsequent biochemical assays. At these time points, a ~30% inhibition of cell viability was observed compared with the control.

### 3.2. KIOM-2015EW Significantly Decreased Pro-Inflammatory Cytokine Expression in HOS-Exposed HCECs

**Figure 2:**
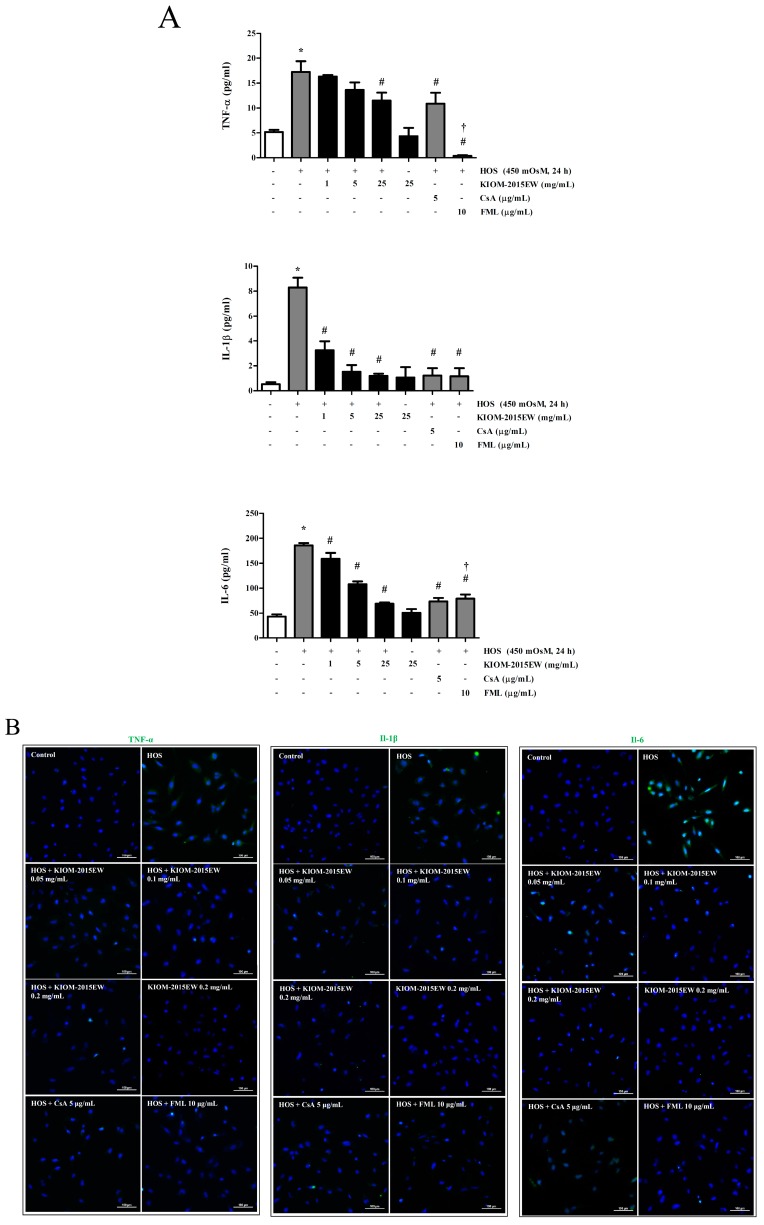

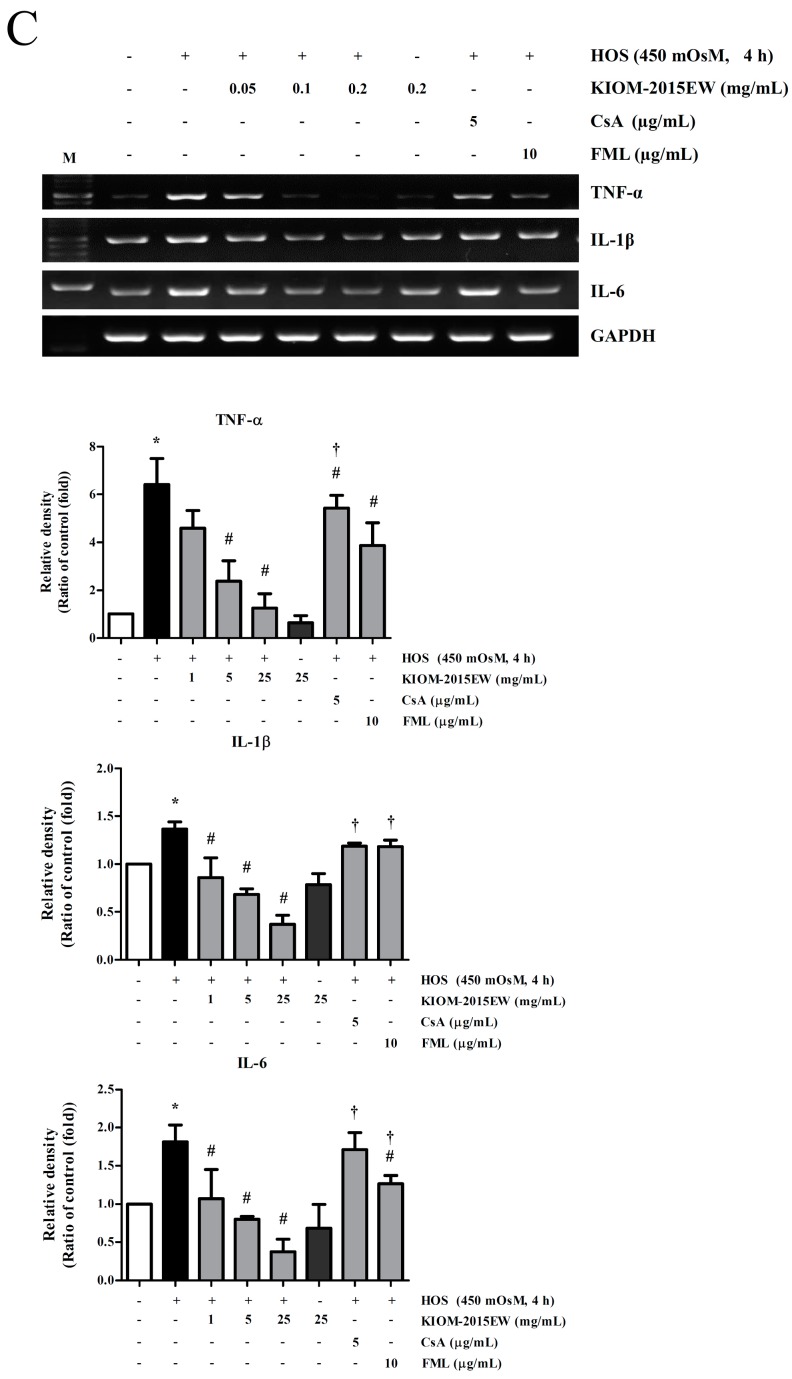
The anti-inflammatory effect of KIOM-2015EW on proinflammatory cytokines (tumor necrosis factor (TNF)-α, interleukin (IL)-1β and IL-6) in HOS-induced HCECs. HCECs were cultured in isomer (312 mOsM) medium, switched to hyperosmotic medium (450 mOsM) alone or in the presence of different concentrations of KIOM-2015EW for 24 h, and protein levels were estimated by enzyme-linked immunosorbent assay (ELISA) (**A**); and immunofluorescence staining (IF) (**B**); or 4 h to evaluate mRNA level by reverse transcription-polymerase chain reaction (RT-PCR) (**C**). CsA and FML were used as positive anti-inflammatory drugs. Data are summarized as mean ± SD from three independent experiments. HOS, hyperosmolar stress; CsA, cyclosporine A; FML, fluoremetholone. * *p* < 0.05 vs. control, # *p* < 0.05 vs. HOS, † *p* < 0.05 vs. KIOM-2015EW (25 μg/mL). The full size blot is shown in Supplementary Figure S1.

Previous studies have demonstrated that HOS significantly increases pro-inflammatory cytokine expression, and the present study confirmed these findings. The TNF-α, IL-1β, and IL-6 concentration was 4.84 ± 0.67, 0.52 ± 0.16, and 43.28 ± 4.33 pg/mL, respectively, in HCECs in the isomolar condition, as evaluated by ELISA (Figure 2A). Hyperosmotic medium (450 mOsM) increased the expression to 15.78 ± 0.40, 8.30 ± 0.78, and 186.24 ± 4.78 pg/mL, respectively. Interestingly, treatment with 0.05, 0.1, and 0.2 mg/mL KIOM-2015EW for 24 h significantly reduced pro-inflammatory cytokine production in a dose-dependent manner. Treatment with anti-inflammatory drugs (CsA or fluoremetholone (FML)) also reduced cytokine production, but their effects were not as marked as that of KIOM-2015EW.

Immunofluorescence staining was performed to detect TNF-α, IL-1β, and IL-6 expression in HCECs. The immunoreactivity of control HCECs to these inflammatory markers was weak (Figure 2B). Positive-staining increased markedly when the cells were exposed to HOS for 24 h, and incubation with KIOM-2015EW attenuated the increased reactivity.

To investigate whether KIOM-2015EW affects pro-inflammatory cytokine expression, the mRNA levels of TNF-α, IL-1β, and IL-6 were determined in HOS-stimulated HCECs. Treatment with 450 mOsM medium increased TNF-α, IL-1β, and IL-6 mRNA expression by 6.41 ± 1.88, 1.36 ± 0.25, and 1.81 ± 0.38 fold, respectively, compared with the control (312 mOsM; Figure 2C). Cytokine expression decreased by 4.58 ± 1.29, 0.86 ± 0.71, and 1.07 ± 0.65 fold, respectively, in HCECs at 450 mOsM with the addition of 0.05 mg/mL KIOM-2015EW. Expression further decreased by 2.37 ± 1.48, 0.69 ± 0.20, and 0.80 ± 0.07 fold, respectively, upon treatment with 0.1 mg/mL KIOM-2015EW and by 1.24 ± 1.04, 0.37 ± 0.33, and 0.37 ± 0.29 fold, respectively, with 0.2 mg/mL KIOM-2015EW. This result suggested that KIOM-2015EW was more effective than the indicated drugs.

### 3.3. KIOM-2015EW Blocked Apoptosis Due to HOS-Induced Cytotoxicity in HCECs

**Figure 3:**
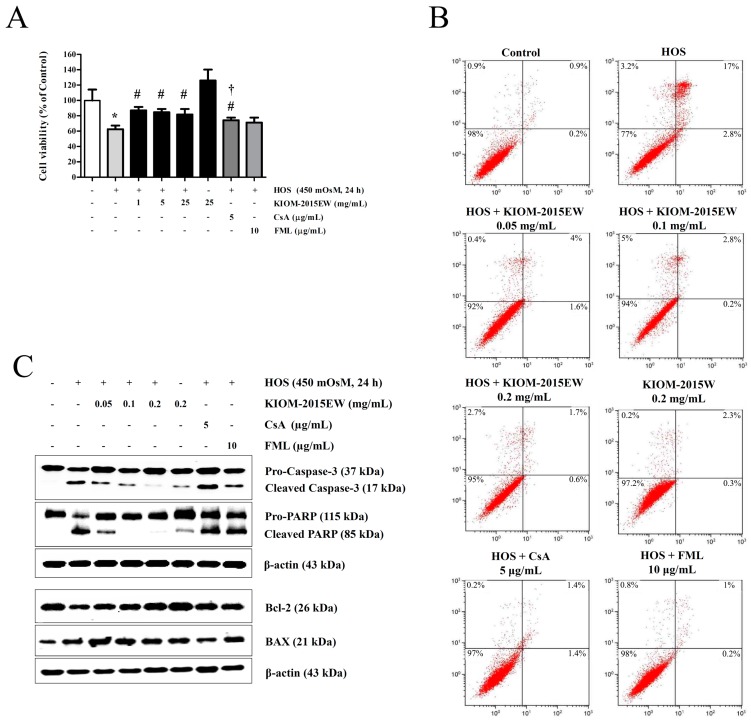
The anti-apoptotic effect of KIOM-2015EW in HOS-induced cell cytotoxicity. HOS-exposed HCECs were treated with KIOM-2015EW for 24 h. CsA and FML were used as positive anti-inflammatory drugs. (**A**) Cell viability was measured by MTT assay; (**B**) After incubation with KIOM-2015EW for 24 h, apoptotic HCECs were stained with Annexin V and propidium iodide (PI) and analyzed by flow cytometry. Cells are represented in the dot plot as healthy (Annexin-V−/PI−, lower-left quadrant), early apoptosis (Annexin-V+/PI−, lower-right quadrant), late apoptosis (Annexin-V+/PI+, upper-right quadrant), and necrosis (Annexin-V−/PI+, upper-left quadrant); (**C**) Expression levels of apoptosis-related markers, including caspase-3, poly (ADP-ribose) polymerase (PARP), Bax, and Bcl-2, were determined by western blot analysis. β-Actin was used as the loading control. The data are presented as the mean ± SD of three independent experiments. * *p* < 0.05 vs. control, # *p* < 0.05 vs. HOS, † *p* < 0.05 vs. KIOM-2015EW (25 μg/mL). The full size blot is shown in Supplementary Figure S2.

Previous studies have indicated that HOS induces HCEC apoptosis through a cytochrome c–dependent pathway, which may be mediated by MAPK signaling [12]. Here, the HCEC survival index was evaluated by MTT assay after treatment of HOS-exposed HCECs with various concentrations of KIOM-2015EW for 24 h (Figure 3A). KIOM-2015EW (0.05–0.2 mg/mL) suppressed HOS-induced cytotoxicity in HCECs. KIOM-2015EW inhibited apoptosis more effectively than CsA and FML.

To quantify the number of apoptotic cells, we performed Annexin V and propidium iodide (PI) for flow cytometry on HOS-exposed KIOM-2015EW-treated HCECs. The percentage of late apoptotic and necrotic cells increased upon HOS-exposure, indicating that both apoptosis and necrosis are major events involved in HOS-induced cytotoxicity in HCECs (Figure 3B). The cell viability in HOS-exposed cells was 77%. In the presence of KIOM-2015EW (0.05–0.2 mg/mL), the cell viability recovered to 92%, 94%, and 95%, respectively.

HCECs were also subjected to apoptosis analysis in the presence/absence of KIOM-2015EW. Caspase 3 and PARP cleavage, and Bcl-2 (anti-apoptotic factor) and Bax were detected with specific antibodies 24 h after hyperosmotic stimulation (Figure 3C). In the western blot analysis, high levels of cleaved caspase 3 and PARP were detected in HOS-exposed cells, whereas KIOM-2015EW-treatment decreased this cleavage in a dose-dependent manner. Bcl-2 expression increased with KIOM treatment, but Bax expression level was not different in HOS- and/or KIOM-2015EW-treated cells (Figure 3C).

### 3.4. KIOM-2015EW Downregulates HOS-Mediated p38, ERK, and JNK Activation in HCECs

**Figure 4:**
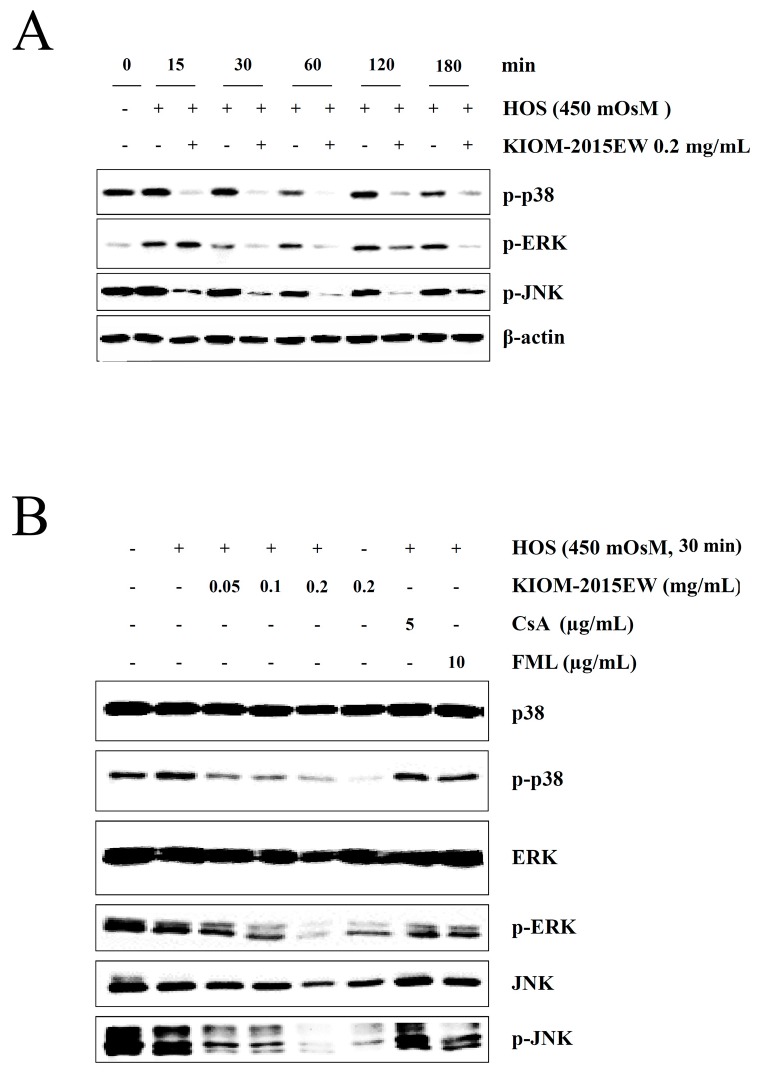
Regulation of Mitogen-activated protein kinase (MAPK) activation by KIOM-2015EW in HOS-induced MAPK phosphorylation. (**A**) HCECs were exposed to HOS for 15, 30, 60, 120, and 180 min, and the expression levels of MAPK proteins, including p38, extracellular signal regulated kinase (ERK) and c-Jun N-terminal kinase (JNK), phospho-p38 (pp38), phospho-ERK (pERK), and phospho-JNK (pJNK), were determined by western blot analysis; (**B**) HOS-exposed HCECs were treated with KIOM-2015EW for 30 min, and the expression levels of pp38, pERK, and pJNK were verified by western blot analysis. CsA and FML were used as positive anti-inflammatory drugs. β-Actin was used as the loading control. Data are presented as the mean ± SD of three independent experiments. The full size blot is shown in Supplementary Figure S3.

Several studies have reported that HOS activates MAPK signaling [12,13,46,47]. Western blotting with phosphor-specific antibodies revealed that the levels of phosphorylated p38, ERK, and JNK increased dramatically in HCECs within 15–120 min of exposure to 450 mOsM hyperosmolar media (Figure 4A). However, in the KIOM-2015EW (0.2 mg/mL)-treatment group, p38, ERK, and JNK phosphorylation was inhibited at the 15 (p38 and JNK) or 30 min (ERK) points, and the inhibition was sustained until 60 min. Based on these results, HOS- and/or KIOM-2015EW-treatment was performed for 30 min in subsequent assays. In KIOM-2015EW (0.05, 0.1, and 0.2 mg/mL)-treated cells, p38, ERK and JNK phosphorylation decreased in a dose-dependent manner (Figure 4B). However, phosphorylation decreased slightly with CsA- and FML-treatment as well. KIOM-2015EW suppressed HOS-induced increases in MAPK signaling activation (Figure 4).

### 3.5. MAPK Inhibition by KIOM-2015EW Blocks HOS-Induced Apoptosis

**Figure 5:**
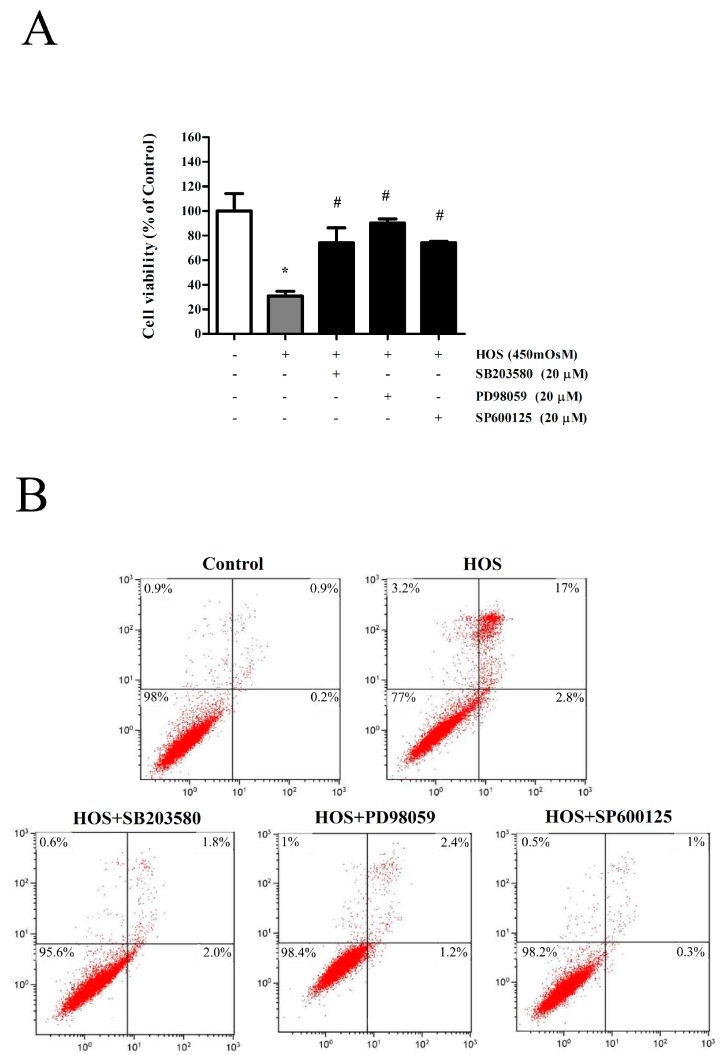
Effect of KIOM-2015EW on MAPK blockade on HOS-induced apoptosis. HOS-exposed HCECs were treated with 20 µM MAPK inhibitors, including SB203580, PD98059, and SP600125, for 24 h. CsA and FML were used as positive anti-inflammatory drugs. (**A**) Cell viability was measured by MTT assay; (**B**) After incubation with MAPK inhibitors, apoptotic HCECs were stained with Annexin V and PI, and analyzed by flow cytometry. The data are presented as the mean ± SD of three independent experiments * *p* < 0.05 vs. control, # *p* < 0.05 vs. HOS.

We investigated whether inhibition of MAPK signaling regulates HOS-induced apoptosis. A p38 MAPK inhibitor (SB203580), an ERK inhibitor (PD98059), and a JNK inhibitor, (SP600125) were used to examine MAPK-related apoptosis. After HOS-exposure for 24 h in the presence/absence of SB203580 (20 µM), PD98059 (20 µM), or SP600125 (20 µM), cell-death was assessed by MTT and fluorescence-activated cell sorting (FACS). HOS-induced cytotoxicity and apoptosis were blocked by co-treatment with SB203580, PD98059, and SP600125, as assessed by Annexin V-FITC and PI staining, (Figure 5A,B), suggesting that MAPK plays an important role in HOS-induced apoptosis. In other words, inhibition of HOS-induced apoptosis by KIOM-2015EW is mediated via MAPK signaling.

### 3.6. KIOM-2015EW Suppresses HOS-Induced NF-κB Activation through p65 Nuclear Translocation Blockage in HCECs

**Figure 6:**
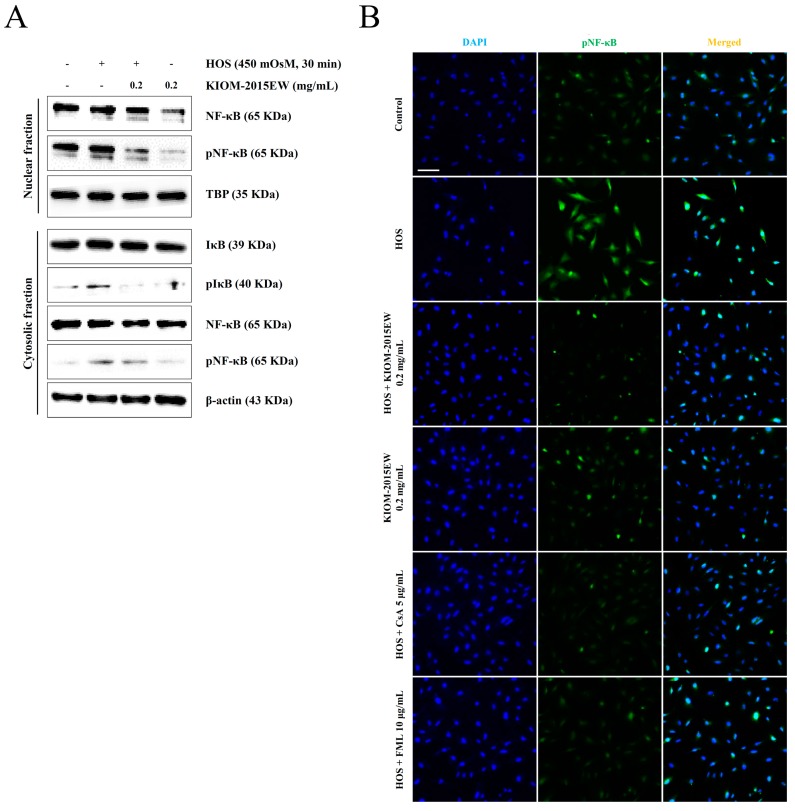
Effects of KIOM-2015EW on the nuclear translocation of NF-κB and phosphorylation of IκB-α in HOS-induced HCECs. (**A**) HCECs were simultaneously exposed to HOS and treated with 0.2 mg/mL KIOM-2015EW for 5 min. Cytosolic proteins were subjected to western blot analysis with the indicated antibodies. β-Actin was used as the internal control for the cytosolic fraction. The numbers represent the average densitometric values relative to β-actin. Data are summarized as mean ± SD from three independent experiments; (**B**) NF-κB p65 activation was examined by immunofluorescence staining. Scale bar, 100 µm. The full size blot is shown in Supplementary Figure S4.

P65 is a key regulator of inflammatory marker genes [48] or pro-apoptotic genes. HOS activates the NF-κB pathway involved the stimulation of IκB kinase (IKK) phosphorylation, IκB-α degradation, p65/RelB NF-κB translocation to the nucleus, and transcriptional activation of NF-κB target genes [49]. Because NF-κB is a major transcription factor that regulates protein expression, we investigated whether KIOM-2015EW inhibits NF-κB release from IκB-α and/or its subsequent translocation from the cytosol to the nucleus. Western blotting using cytosolic factions revealed that IκB phosphorylation was markedly decreased after KIOM-2015EW-treatment (Figure 6A). Immunofluorescence staining revealed that KIOM-2015EW inhibited HOS-induced nuclear translocation of NF-κB (Figure 6B), suggesting that KIOM-2015EW regulates the HOS-mediated IκB–NF–κB circuit.

### 3.7. Identification of the Main Components in KIOM-2015EW Using HPLC

**Figure 7:**
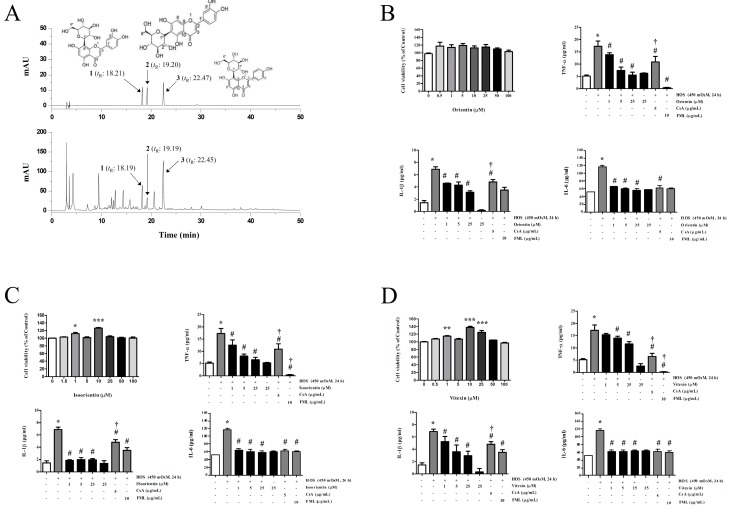
High- performance liquid chromatography with diode array detection (HPLC-DAD) chromatograms of three compounds in KIOM-2015EW and the anti-inflammatory effect on proinflammatory cytokines (TNF-a, IL-1β and IL-6) in HOS-induced HCECs. Orientin (1); isoorientin (2); vitexin (3) in standard mixture and KIOM-2015EW (**A**) were identified at wavelengths of 280 nm. HCECs were cultured in isomer (312 mOsM) medium, switched to hyperosmotic medium (450 mOsM) alone or in the presence of different concentrations of orientin (**B**), isoorientin (**C**) and vitexin (**D**) for 24 h. cell viability measured by CCK-8 assay and protein levels were estimated by enzyme-linked immunosorbent assay (ELISA). CsA and FML were used as positive anti-inflammatory drugs. Data are summarized as mean ± SD from three independent experiments. HOS, hyperosmolar stress; CsA, cyclosporine A; FML, fluoremetholone. * *p* < 0.05 vs. control, # *p* < 0.05 vs. HOS, † *p* < 0.05 vs. KIOM-2015EW (25 μg/mL).

According to the maximum absorption of the standards, a UV detector was set at 280 nm for HPLC analysis of three compounds, including orientin, isoorientin, and vitexin. The HPLC chromatograms of the standard mixture and KIOM-2015EW extract are presented in Figure 7A,B. By comparing the retention times and ultraviolet (UV) spectral data with the standard compounds, the peaks 1, 2 and 3 of KIOM-2015EW were identified as orientin, isoorientin and vitexin, respectively. The mixed standards were indicated at the retention time of 18.21 min (1); 19.20 min (2) and 22.47 min (3) in the chromatogram. These compounds were identified in the KIOM-2015W at similar retention times (1, 18.19 min; 2, 19.19 min; 3, 22.45 min). Other major peaks of retention time were not able to be identified. Therefore, we are currently separating these peak for nuclear magnetic resonance (NMR) analysis.

### 3.8. Orientin, Isoorientin and Vitexin Reduced Level of HOS-Induced Pro-Inflammatory Cytokines

To examine the anti-inflammatory effects of three major compounds, we identified levels of pro-inflammatory cytokines (TNF-α, IL-1β and IL-6) in HOS-treated HCECs (Figure 7). The levels TNF-α, IL-1β and IL-6 were significantly reduced by orientin, isoorientin and vitexin (1, 5 and 25 μM) treatment in HOS-induced cytokines. The anti-inflammatory effects of these compounds were confirmed to be in a dose dependent manner (Figure 7B–D).

## 4. Discussion

DES is caused partly by increased osmolarity of the tear film, resulting in inflammation and subsequent cell damage [1,50]. In both animal- and cell-based studies, HOS induced the production and expression of pro-inflammatory cytokines in the ocular surface cells [13,14,19,20].

Increasing evidence suggests that the expression of inflammatory mediators (IL-6, IL-1β, and TNF-α) on the ocular surface may play a role in DES pathogenesis [8,51,52]. Changes in tear composition, including increased cytokine, chemokine, metalloproteinase, and T cell numbers in the conjunctiva are reported in dry eye patients and animal models. This inflammation is partly responsible for eye irritation, ocular surface epithelial disease, and altered corneal epithelial barrier function in dry eye [53]. There are several anti-inflammatory therapies for dry eye that target one or more inflammatory mediators/pathways (corticosteroids, cyclosporine, tetracyclines and their derivatives, and essential fatty acids) [53].

To date, the only Food and Drug Administration (FDA)-approved drug for DES treatment in the United States is CsA (Restasis^®^, Allergan, Irvine, CA, USA), which is purported to act partly by inhibiting T-cell-stimulated cytokines by binding to the nuclear proteins required for T-cell activation [54,55]. Topical corticosteroids can be used to ameliorate DES, but their long-term use is limited owing to side effects such as increased intraocular pressure and cataract formation [54,55].

Recent studies have reported that many natural products (blueberry, green tea, curcumin, quercetin, and resveratrol) have anti-inflammatory effects on DES or ocular disease [56,57,58,59]. In oriental medicine, herbal roots, bark and branches of the genus *Acer* (maple) have been used as a medicine for their antioxidant, antitumor, and anti-inflammatory activity [57]. Over the years, medicinal plants of the Acer genus have been shown to treat rheumatism, bruises, eye disease, and pain, in addition to detoxification [57]. However, the traditional uses of these plants have been recorded primarily in local herbal books or have been passed down orally from one generation to another [57]. The medicinal use of this genus, compared to its ornamental and food uses, must be investigated further as it is widespread and of known therapeutic efficacy [57]. Little is known about the bioactivity and potential clinical implications of KIOM-2015EW, a natural-substance-derived herbal product, on the health of the human eye. Here, we present evidence that KIOM-2015EW may have beneficial effects on eye and ocular surface diseases. We explored the beneficial effects of KIOM-2015EW using an in vitro HCEC culture model of HOS-induced dry eye. Our findings demonstrate that HOS induces inflammation and that KIOM-2015EW inhibits HOS-induced pro-inflammatory cytokines (TNF-α, IL-1β, and IL-6) in HCECs, with comparable efficacy and potency as corticosteroids and CsA.

Several studies have reported that MAPK signaling is activated by HOS [12,13,38]. HOS induces HCEC apoptosis through a cytochrome c-mediated death pathway, which may be mediated by JNK and ERK1/2; JNK and ERK mediate IL-1β, TNF-α, and IL-8 induction in human limbal epithelialcells [12]. The MAPK cascades are serine/threonine-specific kinases that play key roles in regulating proinflammatory cytokine production [60]. Hyperosmolarity-induced cytokine release in HCECs is mediated via multiple MAPKs, including ERK, JNK, and p38 [13,14]. We demonstrated that these cytokines are stimulated by HOS and inhibited by KIOM-2015EW, similar to the effects of CsA and FML.

NF-κB activation by HOS has been reported previously. HOS induces nuclear translocation of NF-κB in HCECs and cultured cardiomyocytes with dichotomic actions on caspase activation and cell death [49,61]. Moreover, the transcription factors NF-κB and AP-1 play a major role in the induction-inflammation-related genes (cytokines, cytokine receptors, chemotactic proteins, and adhesion molecules) [62]. Here, we demonstrated that KIOM-2015EW inhibits HOS-induced NF-κB transcriptional activity, indicating that the anti-inflammatory effects of KIOM-2015EW are mediated via this signaling pathway. 

Among the compounds identified in KIOM-2015EW, the major flavonoids were determined to be C-glycosylflavones (orientin, isoorientin and vitexin). Orientin, isoorientin and vitexin are components of many natural plant extracts, and many other researchers have proved their anti-inflammatory [63,64,65], anti-cancer [66,67,68,69] and antioxidant [70] effects. These results suggest that these compounds in KIOM-2015EW may contribute to its anti-inflammatory properties.

These findings suggest that the dry eye treatment strategy should include a combination of agents that protect the eye from inflammatory injuries, in addition to simple tear-related interventions. The development of a natural product for dry eye prevention and treatment would benefit many patients with eye disorders.

In summary, the current study demonstrated that the natural substance, KIOM-2015EW, effectively protects HCECs from HOS-induced inflammation by reducing inflammatory cytokine production through the regulation of MAPK signalling and NF-κB translocation to the nucleus. Our findings for the first time suggest the potential benefits of a natural plant extract on ocular surface disorders, such as DES.

## Figures and Tables

**Table 1:** Primer sequences, annealing temperature, and cycles used for reverse transcription polymerase chain reaction (RT-PCR).

Primers				
Gene Name	Sequence		Tm. (°C)	Cycles
*TNF-α*	F	5′-GCGGTGCTTGTTCCTCAG-3′	58	40
	R	5′-CTGGCAGGGGCTCTTGAT-3′		
*IL-1β*	F	5′-TCAGCACCTCTCAAGCAGAA-3′	58	30
	R	5′-ATCAGAATGTGGGAGCGAATG-3′		
*IL-6*	F	5′-CAATCTGGATTCAATGAGGAGACTT-3′	58	30
	R	5′-CCATTAACAACAACAATCTGAGGTG-3′		
*GAPDH*	F	5′-GAAGGTGAAGGTCGGAGT-3′	56	35
	R	5′-GAAGATGGTGATGGGATTTC-3′		

TNF-α, tumor necrosis factor-α; IL, interleukin; GAPDH, glyceraldehyde 3-phosphate dehydrogenase; F, forward; R, reverse.

## Supplementary Materials

The following are available online at http://www.mdpi.com/2072-6643/10/3/282/s1, Figure S1: KIOM-2015EW regulates mRNA of proinflammatory cytokines in HOS-induced HCECs. This is a full length image of the cropped blots presented in the Figure 2C, Figure S2: KIOM-2015EW reduces HOS-induced apoptotic cell death in HCECs. This is a full length image of the cropped blots presented in the Figure 3C, Figure S3: KIOM-2015EW regulates HOS-induced MAPK phosphorylation. (A) is an uncropped image for Figure 4A. (B) is an uncropped image for Figure 4B, Figure S4: KIOM-2015EW regulates the phosphorylation and nuclear translocation of NF-κB in HOS-induced HCECs. This is a full length image of the cropped blots presented in the Figure 6A.
