# Supplementary material for: Anti-Inflammatory and Anti-Apoptotic Effects of Acer Palmatum Thumb. Extract, KIOM-2015EW, in a Hyperosmolar-Stress-Induced In Vitro Dry Eye Model
Source: Nutrients. 2018 Feb 28;10(3):282. doi: 10.3390/nu10030282 (PMC5872700; doi:10.3390/nu10030282)
Supplement: Supplementary file 1 [file nutrients-10-00282-s001.pdf]

nutrients

Supplementary

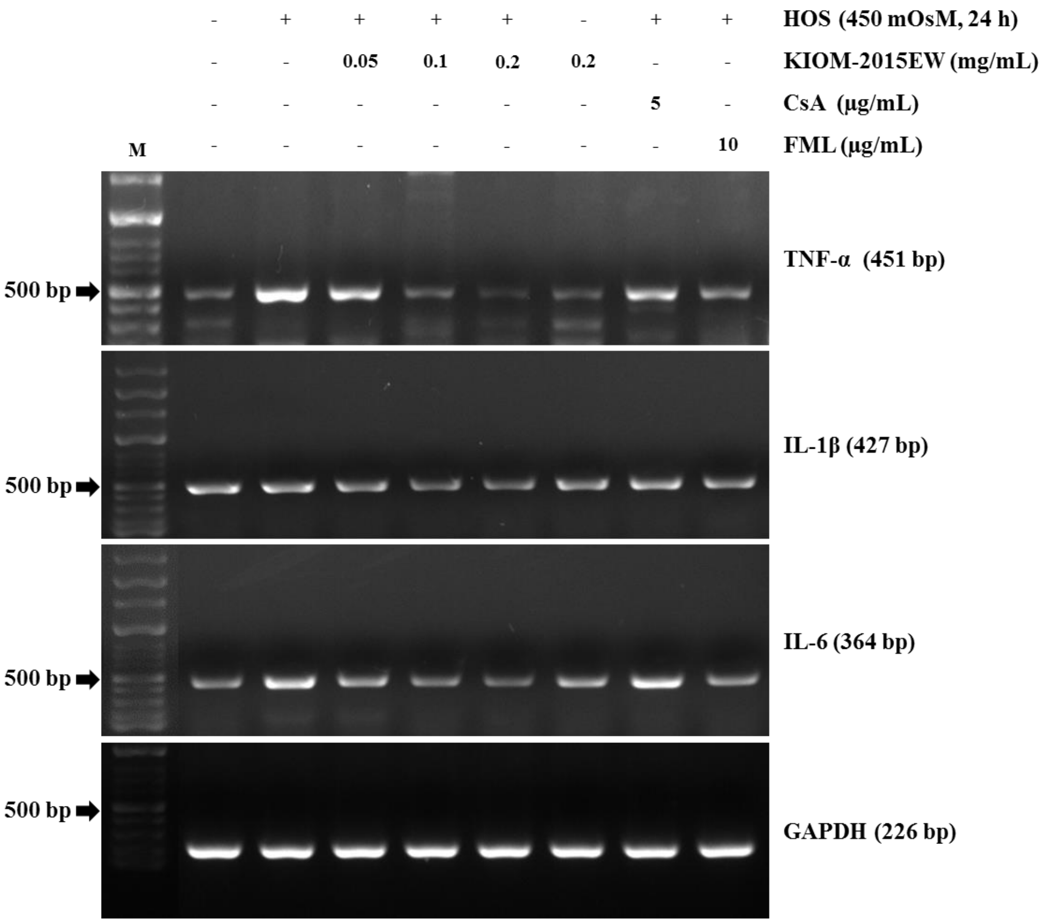

Figure S1. KIOM-2015EW regulates mRNA of proinflammatory cytokines in HOS-induced HCECs. This is a full length image of the cropped blots presented in the Figure 2C.

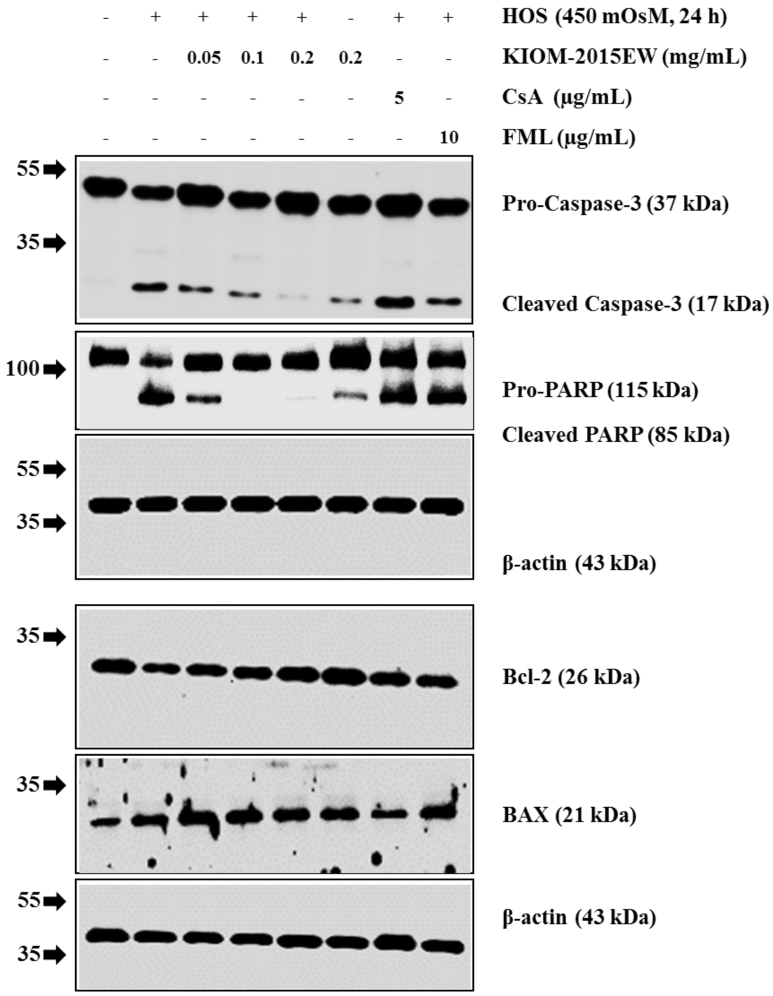

Figure S2. KIOM-2015EW reduces HOS-induced apoptotic cell death in HCECs. This is a full length image of the cropped blots presented in the Figure 3C.

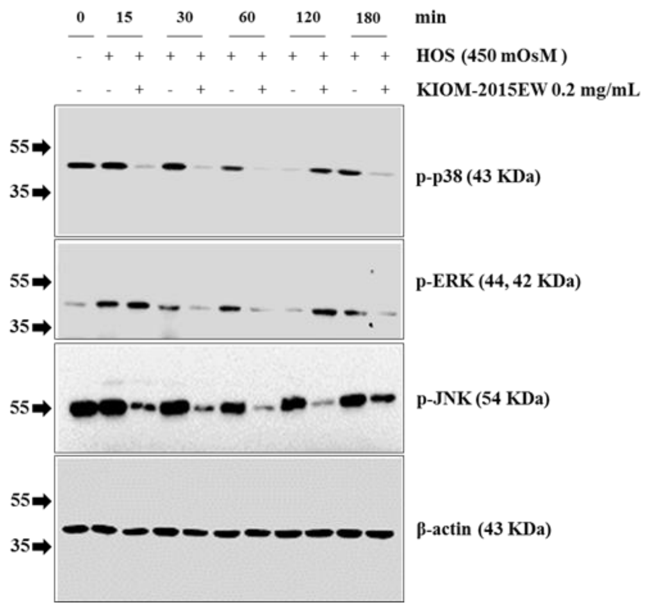

(**A**)

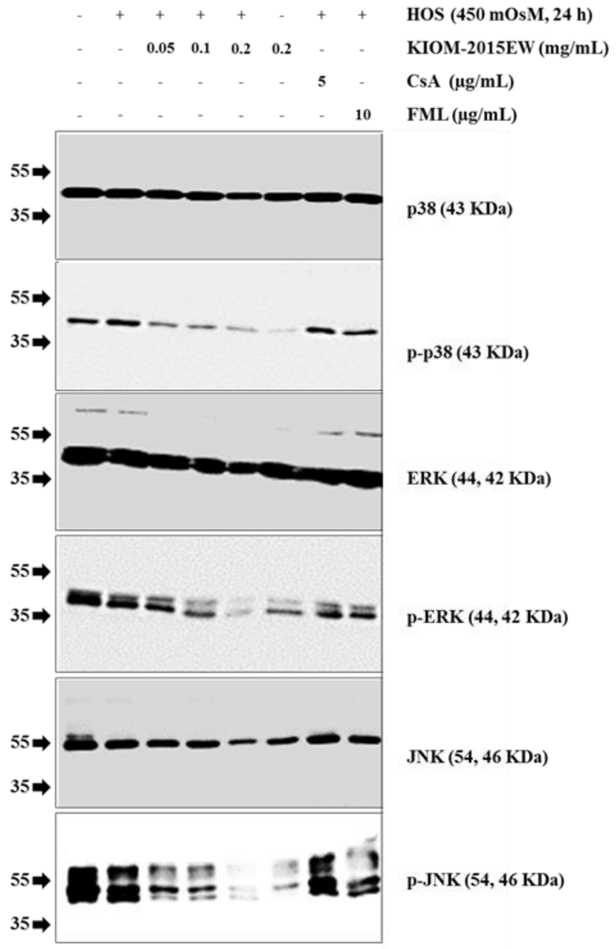

(**B**)

Figure S3. KIOM-2015EW regulates HOS-induced MAPK phosphorylation. (**A**) is an uncropped image for Figure. 4A, (**B**) is an uncropped image for Figure. 4B.

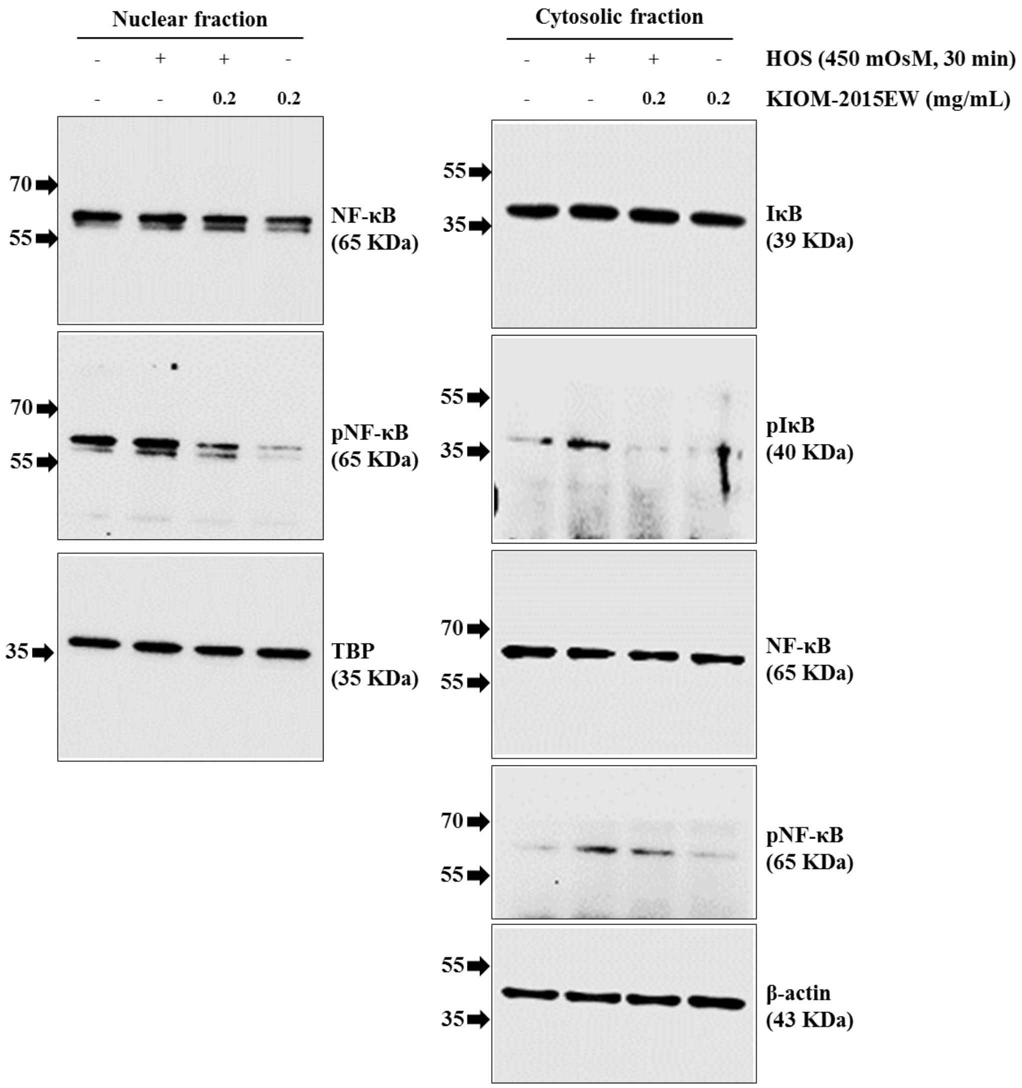

Figure S4. KIOM-2015EW regulates the phosphorylation and nuclear translocation of NF-κB in HOS-induced HCECs**.** This is a full length image of the cropped blots presented in the Figure 6A.

© 2018 by the authors. Submitted for possible open access publication under the terms and conditions of the Creative Commons Attribution (CC BY) license (http://creativecommons.org/licenses/by/4.0/).
